# The inclusion of the gender perspective in oncology research with Spanish participation

**DOI:** 10.1016/j.heliyon.2024.e30043

**Published:** 2024-04-27

**Authors:** Rut Lucas-Domínguez, María Aragonés González, Andrea Sixto-Costoya, Emmanuel Ruiz-Martínez, Alonso Alonso-Arroyo, Juan Carlos Valderrama-Zurián

**Affiliations:** a Grupo UISYS. Departamento de Historia de la Ciencia y Documentación, Facultad de Medicina y Odontología, Universitat de Valencia, Spain; b Unidad asociada al Instituto Interuniversitario de Investigación Avanzada sobre Evaluación de la Ciencia y la Universidad (INAECU), UC3M-UAM3, Spain; c CIBERONC, Valencia, Spain; d Universitat d’Alacant, Grupo de Investigación sobre Trabajo Social y Servicios Sociales (GITSS), Spain; e Departamento de Trabajo Social y Servicios Sociales, Facultad de Ciencias Sociales, Universitat de València, Spain

**Keywords:** Gender perspective, Gender balance, Oncology research, Spain, Cancer

## Abstract

The gender perspective is important for a better diagnosis and treatment of diseases, especially in the field of oncology. This study aimed to analyse the gender approach in scientific articles in the field of oncology by studying the gender composition of the authorship of papers and the gender inclusion in the research carried out. A bibliographic search of articles and reviews signed by at least one Spanish institution published between 2010 and 2019 was carried out using the Science Citation Index Expanded database in the Oncology category. A total of 7523 studies were classified according to the gender composition determined by the author's name and a randomised sample was used to evaluate the inclusion of gender perspectives using a checklist. This study revealed a lack of gender parity in the authorship of oncology publications involving Spanish participation. Papers without author gender parity were eight times higher than papers with parity and showed a greater presence of male than female authorship (58 % versus 31 %). Regarding the introduction of the gender perspective, a negative response of 68 % referring to compliance with the entire checklist was obtained, and only a fifth of the articles presented gender balance in the study sample. Moreover, there is a positive correlation between gender parity in authorship and gender perspective integration in published research. In conclusion, there is a great need to advance the inclusion of gender perspectives in cancer research to overcome gender bias and promote better prevention, detection, and intervention for cancer.

## 1 Introduction

Cancer is a major global health concern. In 2020, an estimated 19.3 million new cases of cancer were diagnosed, and nearly 10 million people died, of which approximately 4.5 million were women [1]. In contrast to the increase in the incidence of the disease, mortality has decreased by 18 % globally since 1990 due to advances in research and access to treatments, as well as to changes in habits and environmental factors resulting from public prevention policies. The incidence of cancer as a disease has unique aspects, both in terms of sex and gender [2,3]. Gender refers to socially constructed characteristics, whereas sex refers to biological or physiological characteristics [4]. In this sense, research should consider both sex and gender during the analysis of trends, diagnosis, prognosis, therapeutics, and the frequency of each tumor as well as other sociocultural and economic dimensions (age, place of origin, income level, ethnic group, etc.) [5]. Although the relevance of the sex variable is easier to visualize both in cancer and in other studies related to health sciences, the importance of gender is underlying and often underestimated. In cancer research, an example of the relevance of the gender variable can be seen when looking at incidences by tumor type. With regard to lung cancer, the incidence and mortality rates in women have increased over the years [6]. The reasons for this increase cannot be explained without taking into account the gender factor, which helps to understand how the increase in smoking-related risk behaviour among women is linked to progressive but constant changes in socialisation patterns, women's independence, integration into working life and the abandonment of traditional roles, especially among younger women [7]. Another example, in this case of how public policies with or without a gender perspective could influence cancer is around breastfeeding facilities (in terms of time dedication, infrastructures and other issues), as it has been shown to be a protective factor for breast cancer. Finally, it was observed that in many cultures there is a double stigmatization when women suffer from cancer due to both, the feeling of guilt for not being able to take care of their assigned role as caregivers and the difficulties or discrimination in the professional field [8].

This implies that several differentiating factors between women and men must be considered for early detection, diagnosis, and treatment in oncology [9]. For these reasons, gender inclusion in oncology is of great relevance and represents an essential approach in recognising the differences of the impact of the disease on individual women and men [10], in addition to ensuring adequate prevention and intervention.

To implement an adequate gender approach, two fundamental aspects are necessary: first, the participation of all genders in the research team, as there is a positive correlation between innovativeness, competitiveness, and the gender equality index in a country [11]. The second is the correct integration of the gender perspective in all stages of research, starting with the team composition, the study design, the methodology and finally the communication of the results [12]. In the health sciences, the gender gap, perceived as an inequality in the levels of participation and access, continues to persist in the professional and scientific fields [13], as well as gender and sex bias in research [14], understood as a lack of sex-disaggregated data or a lack of inclusion of a gender perspective.

The correct integration and inclusion of a gender perspective is even more necessary when physiological and sociocultural characteristics are determinants in research. However, historically, in the health field, it has been assumed that the male model, both in terms of sex and gender, can be taken as a standard pattern, and that its results can be extrapolated to the entire population [9,15]. When data are not sex-disaggregated, it is more difficult to identify real and potential inequalities. Therefore sex-disaggregated data are necessary for subsequent effective gender analyses [4]. In this regard, unequally disaggregated samples have potentially adverse effects that go far beyond the quality of the study itself, as the analysis performed on one sex in the preclinical and clinical phases is then translated and used in patients of the opposite sex in public health research [14]. This reality has led to the underrepresentation of women and non-binary identities in human clinical trials and research, even in population groups used as drug recipients. Consequently, in these underrepresented groups, side effects were not evident until the drugs became commercially available [12,16]. Similarly, gender biases in oncology research lead to an underrepresentation of women in the area of oncology, which may influence the results obtained from clinical investigations and consequently lead to unexpected undesired consequences [9,17]. Therefore, to obtain reproducible and accurate results, sex as a biological variable should be integrated in all basic, preclinical, and clinical research [18,19] and gender as a psychosocial variable, if the type of research requires it. Previous studies showed that sex-related reporting increased from 59 % to 67 % in clinical studies from 2000 to 2016 and from 36 % to 69 % in public health research. However, in biomedical research, sex remains largely underreported (31 % in 2016) [14]. In relation to the consideration of gender, understood in health studies as the critical examination of how social differences affect the gender of belonging, scientific evidence shows that research is still scarce and not widespread in its approach [20].

The aim of this study was to evaluate the integration of the gender perspective in Spanish cancer research in the last decade by analysing 1) the gender gap in the authorship of publications, 2) the gender bias in the scientific content described in published research, and 3) whether there is a relationship between the parity of the signatories and gender inclusion in the research conducted.

## 2 Methods

### 2.1 Evaluation of gender composition in the authors of publications

A bibliographic search was performed using the Web of Science (WoS) Core Collection - Science Citation Index Expanded (SCIE) database for articles and reviews in the Oncology category (WoS Category field = Oncology), which included Spanish institutions. The term Spain was searched in the Address field, and the search was limited to the period of 2010–2019. A total of 12,474 retrieved records were exported to the Microsoft Access database. Subsequently, author names and the institution's country were standardised. Genderize. io software was used to identify and assign genders based on the name and country of each author (obtained through the WoS Address field). Subsequently, we manually reviewed the gender assignment process to verify that it was completed correctly. Through this procedure, we only considered as “valid” for our study the papers where the gender of all authors could be identified, which was a total of 7523 papers (60.3 %). Papers were then assigned to the following groups: all female authors, all male authors, equal numbers of male and female authors, mixed authorship with a greater female presence, and mixed authorship with a greater male presence.

## 3 Analysis of the gender perspective in the content of scientific articles

Thirty articles were randomly selected from each of the aforementioned groups to analyse the gender perspectives in the content of the published research. We selected articles with more than three authors and excluded those on cancer forms that physiologically affect only one sex, such as prostate, uterine, testicular, or ovarian cancer. The remaining cancer types were all included, regardless of the degree of incidence in women or men.

For the analysis of gender approach in the content of the papers included in our study, we considered that a gender balance exists when there is a 40–60 % representation of both sexes, i.e., the ratio was calculated as the number of women divided by the number of men in the sample, and should be between 0.66 and 1.5, following the concept description of “gender balance” published by the Directorate-General for Research and Innovation [21]. Furthermore, the same institution described “gender parity” as a 50:50 balance in the number or proportion of women and men, which corresponds to a ratio of 1 [21]. Gender parity was defined in this study when the authorship of the papers had equal numbers of male and female authors.

Subsequently, a proprietary questionnaire on the inclusion of the gender approach composed of 12 variables (Table 1) was developed based on the questionnaires “Simple checklist for sex-sensitive and gender-sensitive research” [22] and the “Checklist for gender in research” [23]. Likewise, a document agreed upon by all the authors was designed to ensure the appropriate use of the criteria to be followed by the researchers responsible for the evaluation: a social worker (AGM) and a nurse (RME) with specialisation in gender studies and gender perspective. In the event of disagreement between the results in any of the items analysed (20 %), the study was re-evaluated by the rest of the authors until a consensus was reached, and what was finally considered was the assessment of the majority of authors. A gender perspective was not considered present when there was only a sociodemographic description of the sample. Finally, a descriptive study of the questionnaire and each of the 12 variables analysed was performed. In this regard, a percentage calculation based on the fulfilment (positive) or not (negative) of the variables 1 to 12 was calculated. In case that the papers whose variable information was not specified in the article either because the type of study did not involve the use of that population cohort or the sex of the participants was not defined, these were treated as “not-applicable”.

**Table 1:** Evaluation of inclusion gender perspective in health research.

	NON	PARITY	PARITY		*Chi-square*	*df*	*p*
	NO	YES	NO	YES			
V1. Was the consideration of the sex or gender variable relevant to the research?	68.3	31.7	50.0	50.0	3.530	1	0.060
V2. Did the study design allow for investigation of sex and/or gender differences?	73.3	26.7	60.0	40.0	2.058	1	0.151
V3. Was the primary outcome analysed according to the sex or gender variable?	69.2	30.8	46.7	53.3	5.318	1	0.021
V4. Were the implications of the research for women and men inserted into the discussion?	89.1	10.9	80.0	20.0	1.774	1	0.183
V5. If the research involves humans as research objects, has the relevance of gender to the research topic been analysed?	85.3	14.2	70.0	30.0	4.199	1	0.040
V6. Have you reviewed the literature and other sources related to gender differences in the research field?	80.8	19.2	83.3	16.7	0.099	1	0.753
V7. Does the methodology ensure that (possible) gender differences will be investigated: that sex/gender-differentiated data will be collected and analysed throughout the research cycle and form part of the final publication?	76.5	23.5	61.1	18.8	0.169	1	0.681
V8. Does the proposal explicitly and comprehensively explain how gender issues will be handled (e.g., in a specific work package)?	100.0		100.0				N/A
V9. Have possible differential outcomes and impacts of the research on women and men been considered?	85.8	14.2	80.0	20.0	0.629	1	0.428
V10. Are questionnaires, surveys, focus groups, etc. Designed to disentangle potentially relevant sex and/or gender differences in your data?	100.0		100.0				N/A
V11. Are the groups involved in the project (e.g., samples, test groups) gender-balanced?	60.9	39.1	77.3	22.7	1.923	1	0.165
V12. Are data analysed according to the sex variable, and are other relevant variables analysed with respect to sex?	74.2	25.8	43.3	56.7	10.486	1	0.001

Parity refers to the composition of the authorship team. Chi-square of Pearson's value (Chi-square); degrees of freedom value (df); p-value (p). *p < 0.05 was considered statistically significant.

## 4 Thematic analysis through keywords

Keywords were extracted from the 150 articles analysed, and these were classified according to cancer typology and anatomical location according to the National Cancer Institute (NCI) classification [24]. Subsequently, the total sample of keywords from the 150 articles was evaluated by frequency and according to the five authorship composition groups established above.

## 5 Relationship between the parity of the signatories and the gender perspective in the research content

The existence of a correlation between the fulfilment of the questionnaire variables and the authorship groups was assessed. Pearson's chi-squared test was performed to assess whether there were statistically significant differences between the 12 variables analysed and author parity. Differences were considered statistically significant at p < 0.05.

## 6 Results

### 6.1 Evaluation of gender composition in the authors of publications

**Fig. 1:**
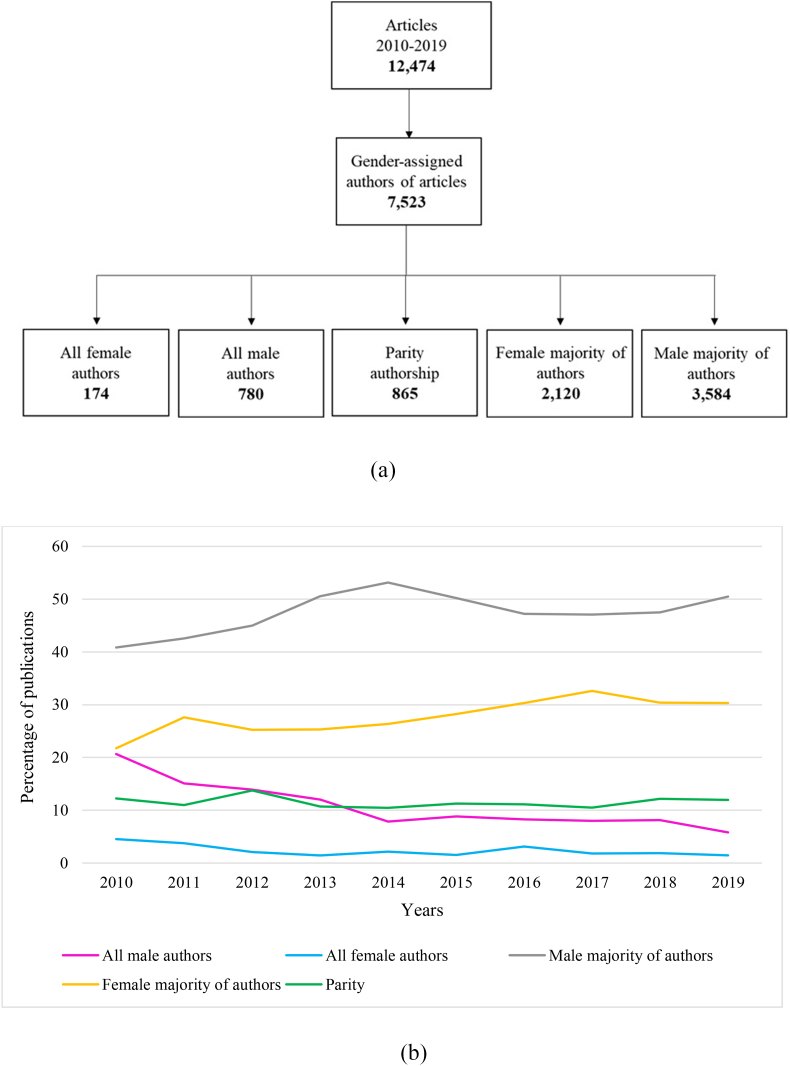
Article author gender groups and number of articles in each group in the study sample (A). Evolution of the percentage of publications by author group over the period 2010–2019 (B).

The 7523 papers were classified according to the five established authorship groups: all female authors (2.3 %), all male authors (10.4 %), equal numbers of male and female authors (11.5 %), mixed authorship with a greater female presence (28.2 %), and mixed authorship with a greater male presence (47.6 %). Papers signed only by women or with a majority of women represented 30.5 %, compared to 58 % of articles signed only by men or with a majority of men (Fig. 1A). Fig. 1B shows the evolution of the number of publications by each group during the study period. Two of the non-homogeneous groups (male majority, female majority) experienced a notable increase in the number of publications, with the parity group remaining stable, and the two homogeneous groups experiencing a decrease.

### 6.2 Analysis of the gender perspective in the content of scientific articles

The analysis of the 150 randomly selected articles according to the 5 authorship groups and the 12 items that comprised the questionnaire on the implementation of the gender perspective in research are presented in Table 1. In general, the overall score of the questionnaire was mostly negative, with a negative response of 68 % for compliance with the total number of items analysed in the 150 articles, compared to 20 % of positive responses. Twelve percent of the responses corresponded to variables whose information was not specified in the article, either because the type of study did not involve the use of a specific population cohort (i.e., a review) or because the sex of the participants was not specified in the sample analysed.

**Fig. 2:**
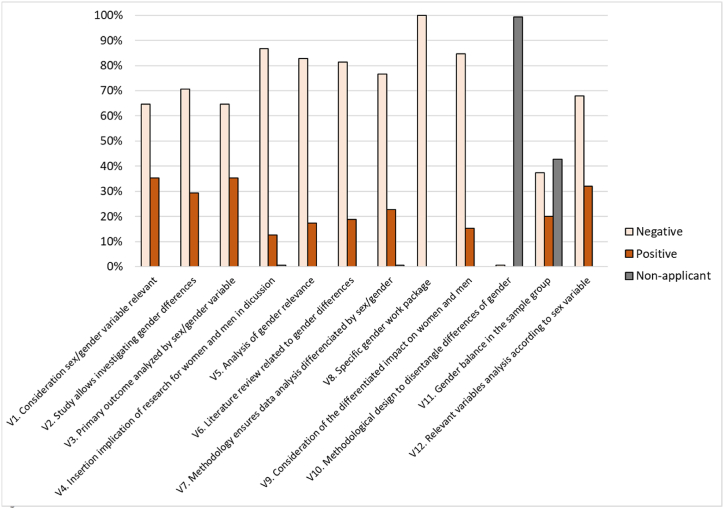
Analysis of the 12 items that make up the questionnaire to evaluate compliance (positive) or not (negative) with the implementation of the gender perspective in health research, being “not applicable” when the document had not studied sex or gender variables.

Analysis of the papers according to each specific item of the questionnaire indicated that only 35.3 % of the articles considered the sex or gender variable relevant (V1) and allowed analysis of the primary outcome of the research described against this variable (V3). However, although 29.3 % of the papers included a design that allowed investigating differences in the sex or gender variable (V2), only 12.7 % contained the implications of the results analysed by sex and/or gender in the discussion (V4) (Fig. 2). It is relevant to mention that most of the articles analysed used the terms “sex” and “gender” interchangeably, but when they considered this variable, it was related to the biological and physiological characteristics of males and females in the vast majority of cases.

Following the order of the variables analysed, 82.7 % of the studies did not include an analysis of the relevance of gender in their research (V5), 81.3 % did not include a review of the literature on gender differences in the specific field of research (V6), and did not consider the impact of the research carried out in a differentiated manner by gender and sex (V9) (84.7 % negative vs. 15.3 % positive). There was no methodology that guaranteed the collection and analysis of data that identified sex and gender differences throughout the studies (76.7 % negative vs. 22.7 % positive) (V7), highlighting the absence in the design of specific work packages and tools for this type of analysis (V8, V10).

Regarding the analysis of the ratio of participant's gender balance (60/40), only 20 % of the articles had gender balance, compared to 37.3 % that did not have gender balance and 42.7 % in which sex or gender was not specified in the sample evaluated (Fig. 2). Interestingly, none of the studies analysed showed gender parity (50/50) in the study sample.

Concerning the thematic analysis of the 150 articles according to the type of cancer investigated and anatomical location, our results showed that the most frequent typologies studied were breast cancer (18.3 %), colorectal cancer (11.8 %), lung cancer (11.1 %), brain cancer (9 %), kidney cancer (8 %), and gastric cancer (8 %). Eleven percent of the evaluated articles did not specify the type of cancer (Supplementary Fig. 1A). These results analysed on the anatomical location or system of neoplasms were similar with on the frequency of tumours affecting the digestive system (21.6 %), followed by the breast (18.3 %), respiratory system (11.1 %), and genitourinary system (8.5 %) (Supplementary Fig. 1B).

### 6.3 Parity of the signatories and the gender perspective in the research content

In the third analysis, compliance was evaluated using the variables of the gender perspective implementation questionnaire according to the composition of the different authorship groups of the articles. We found significant differences between the groups of authors with gender parity (P) (30 studies) and the groups without gender parity (NP) (120 studies) in some of the variables analysed (V3, V5, and V12) (Table 1).

It was found that in variable V3, referring to an analysis of the primary outcome of the research according to the variable sex or gender, 30.8 % of the articles whose authors did not have gender balance met this requirement, while 53.3 % of the articles whose authorship had parity did (Table 1). Similarly, variable V5, referring to research with humans and the analysis of the relevance of gender to the research topic, it was met in 14.2 % of articles without authorship parity; however, this value doubled to 30 % in articles with parity of authors (Table 1). Finally, variable V12, referring to the analysis of the data according to sex and to the analysis of other relevant variables with respect to sex, 25.8 % met this requirement in the group of authors without parity, while this value rose to 56.7 % in the group of authors with parity (Table 1).

**Fig. 3:**
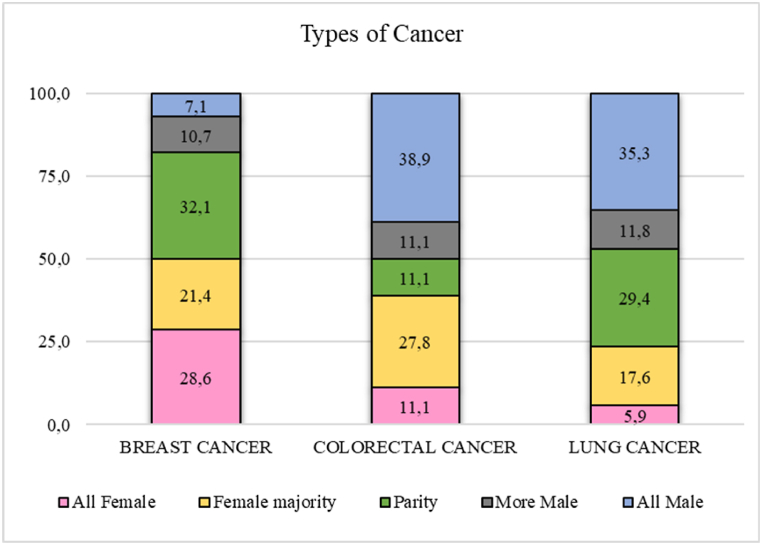
Analysis of the most frequent types of cancer investigated according to the gender composition of the authorship team.

With respect to the analysis of the most frequent types of cancer studied in the random sample of 150 papers according to the aforementioned authorship groups, we found that the group of all-female authors did most research on breast cancer (28.6 %) compared to the group of all-male authors who did most research on colorectal cancer (38.9 %) and lung cancer (35.3 %) (Fig. 3).

## 7 Discussion

The current study in the field of cancer with Spanish participation evaluated the gender gap in the authorship of publications, the gender bias in the scientific content, and the relationship between the parity of the signatories and gender inclusion in the research conducted. Regarding the authorship composition of the derived publications, our results showed first the absence of gender parity, with eight times the number of papers without parity compared to papers with parity. Also, the male presence is still much more frequent than female presence in the research team, both in the groups “all male” versus “all female” (4.5 times); and in the groups “more male” versus “more female” (1.7 times). These results are in line with a report published by the European Commission [21], which shows that the average proportion of women among authors in publications in medical and health sciences was 0.4 in the European Union and 0.3 in the rest of the world (where 0.5 is equal parity) between 2015 and 2019. In a similar way, a large-scale bibliometric study worldwide that included all scientific disciplines showed that women represented an even lower percentage, less than 30 % [25]. Regarding papers with parity in the authorship, it is relevant to mention that even in this apparently “ideal” situation, inequalities between women and men could be also possible to detect due to the signatory positions that they occupy (first and last, which are the most prestigious in health sciences) [[26], [27], [28]]. Moreover, an imbalanced authorship representation does not necessarily indicate gender biases either if women are well represented in the most prestigious positions [29]. Regarding the specific area of oncology, the study by Yalamanchali et al. [30] examining articles of research journals from 3 disciplines in the oncology literature shows that female authorship have increased between 2002 and 2018 in cancer research, being 25.5 % and 31.7 % respectively, but this is still far from parity or even gender balance. In our study it is interesting to note that when the evolution by year is analysed, both groups of papers signed by all female or all male have decreased while those of mixed authorship (female majority and male majority) have increased. This could show that although it is still unequal, there is a trend towards increased collaboration between women and men. Although in our study the analysis of the position of the signer was not carried out, it would be very interesting to do so in the future in order to analyse gender representation in the most prestigious first and last authorship positions and to check whether the decrease in the number of papers signed only by women and only by men also has an impact on the leading positions in authorship.

Regarding the analysis of the inclusion of sex and gender variables, our results showed that we are still far from complying with a scientific methodology that equals the presence of both sexes in all phases of cancer research, as 68 % of studies had a negative compliance with the total number of items required for research with an equal gender approach. In addition, a detailed analysis of each of the variables of the questionnaire showed that none of the studies complied with more than 40 % and that only 20 % of the articles showed gender balance in the sample under study.

The gender perspective in health contemplates that to carry out a complete and effective study, the sex of individuals must be taken into account, as well as an understanding of gender that transcends biological and anatomical aspects [31]. The World Health Organization (WHO) defines gender as the result of socially constructed ideas about a particular sex (behaviours, actions, gender roles, etc.) [32]. In addition, a persistent lack of consideration has been detected in the literature regarding the inclusion of the gender perspective in studies in a transversal and transcendent way to biological sex, a fact that could affect research findings in health science regarding its equal applicability to women and men, and more specifically, in cancer research, which is currently the leading cause of death worldwide [33,34]. Therefore, it is necessary to consider sex and gender variables in the health of women and men, as research results are subsequently implemented in the diagnosis and development of treatments, which inevitably inherit this absence [9,35]. This is even more desirable in oncology, where personalised medicine is becoming increasingly necessary.

Establishing a point of union between the two aspects analysed in this work—the participation of all genders as research personnel and the correct integration of the gender perspective in the content of the published research—a correlation between the incorporation of the gender perspective and publication authorship composition has become clear. According to Sugimoto et al. (2019) [14], gender disparities in scientific teams can affect rigorous and effective medical research. These results are consistent with those of our study, where groups with greater gender parity among their members obtained more positive percentages regarding the inclusion of the gender perspective in their research content.

In addition, the evaluation of the investigated subject matter indicated that it corresponded to the types of cancer with the highest incidence, such as breast cancer (18 % of the total articles in our study), lung cancer (12 %), and colorectal cancer (11 %), or those that generated the highest mortality, such as lung cancer, followed by colorectal cancer, which were the most predominant typologies in our study [1]. Furthermore, our results showed a slightly greater presence of female authorship (50 %) in breast cancer research compared to colorectal cancer (39 % female authorship) and lung cancer (23,8 %). Interestingly, the parity teams were similar for breast cancer and lung cancer papers (30 %).

Despite observing a relationship between the composition of the research team and the inclusion of the gender perspective, it has become evident that in general, the inclusion of the gender variable and its analysis as a sociocultural component is still scarce in oncology research. Along these lines, according to this study, the solution involves analysing sex and gender differences in all stages of a study, which would grant a greater validity and application of the study results for diagnostic and therapeutic interventions in both sexes [36].

This study highlights the need to promote the inclusion of gender mainstreaming in science and the balanced presence of women and men in research teams. This involves the development of improvements in the methodological guides for the inclusion of the gender perspective in all research stages (up to the final scientific publication) that could reduce the gender biases, as well as the training of all the actors involved in the research communication process (from author/s, to the editors and the reviewers) focused on the correct use of the terminology (especially related to “sex” and “gender”).

### 7.1 Limitations

This study analysed papers indexed in the oncology category of the WoS SCIE; therefore, additional oncology papers that were included in other SCIE categories or other databases may not have been retrieved. In this study, the authors used a binary variable for practical reasons since the papers of our sample mainly showed cohorts of patients by male/female sex or animal samples divided into male and female animals.

The statistical package Genderize. io used in this study provides the probability of male or female gender based on a frequently updated database that currently includes more than 200,000 distinct first names from more than 79 countries and languages. Although Genderize. io has been used in prior related work and provides a minimum accuracy of more than 80 % [37,38], it is assumed, as a limitation, the fact that we cannot achieve 100 % accuracy.

Some of the evaluated studies did not mention the number of men and women in the study cohorts because they were reviews. In these cases, it was not possible to calculate the sex ratios. If the cohort studied was a sample composed exclusively of women or men, it was indicated that there was no gender balance, and the ratio equaled 0.

Finally, the content analysis of the sample research showed that 18 % of the articles dealt with breast cancer, which may have introduced a bias in the population studied, which was predominantly female, and in the authorship composition, which was over-represented by women.

## 8 Conclusions

The general results of this study showed that less than half of the articles analysed included the insertion of the sex variable, with even less representation of data disaggregated by sex in the research carried out, which prevents an analysis of the results and the impact of the disease on women vs men. Consequently, the introduction of the gender perspective is still scarce, both in research and in the professional-scientific field, with a male majority of authors signing the published publications and only a tenth of the papers signed in gender parity. Additionally, the benefits of increasing gender and sexual diversity in research teams should be considered, which also leads to better gender and gender inclusion in the research methodology.

## Funding

This work benefited from the assistance of the Spanish Ministry of Equality (MUJER-PI-41-2-ID22), the National R + D + I of the Ministry of Science and Innovation of the Spanish Government (PID2019-108579RB-I00), and the Valencian Regional Ministry of Innovation, Universities, Science, and Digital Society. Generalitat Valenciana (AICO/2020/010; CIAICO/2021/205); the 2015-Networks of Excellence Call (CSO2015-71867-REDT), and the Ministry of Education and Vocational Training (MS21-020).

## Data availability statement

The data generated and used in this study are openly available from the Zenodo.org public repository at https://zenodo.org/records/10040288.

## CRediT authorship contribution statement

**Rut Lucas-Domínguez:** Writing – review & editing, Writing – original draft, Supervision, Project administration, Methodology, Investigation, Funding acquisition, Formal analysis, Data curation, Conceptualization. **María Aragonés González:** Writing – original draft, Methodology, Investigation, Formal analysis, Data curation. **Andrea Sixto-Costoya:** Writing – review & editing, Writing – original draft, Supervision, Resources, Methodology, Formal analysis, Data curation, Conceptualization. **Emmanuel Ruiz-Martínez:** Methodology, Formal analysis, Data curation. **Alonso Alonso-Arroyo:** Writing – original draft, Supervision, Project administration, Investigation, Funding acquisition, Formal analysis, Conceptualization. **Juan Carlos Valderrama-Zurián:** Conceptualization, Funding acquisition, Investigation, Project administration, Writing – original draft, Writing – review & editing.

## Declaration of competing interest

The authors of this study have no interest to declare.
