# Supplementary figures and images for: The inclusion of the gender perspective in oncology research with Spanish participation
Source: Heliyon. 2024 Apr 27;10(9):e30043. doi: 10.1016/j.heliyon.2024.e30043 (PMC11096823; doi:10.1016/j.heliyon.2024.e30043)

**
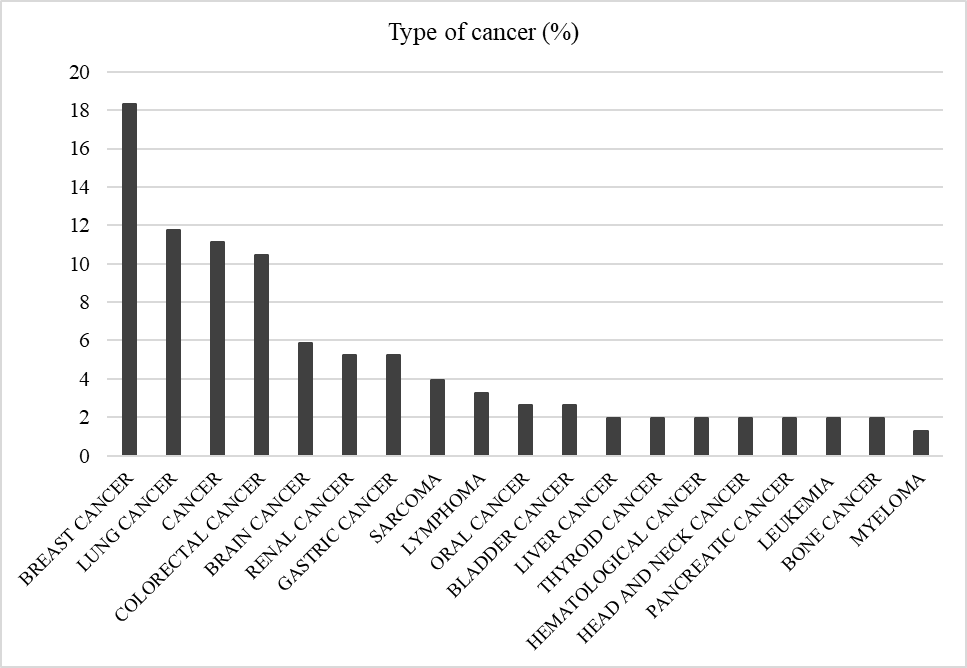
**


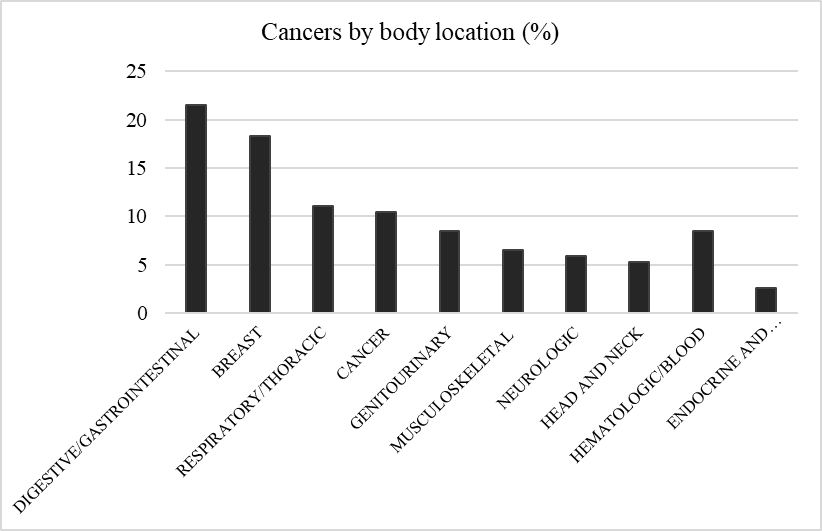
Figure 3. Cancer frequency classified by typology (A) and body location (B).

Supplement: Multimedia component 1 [file mmc1.docx]
